# Comprehensive classification of *TP53* somatic missense variants based on their impact on p53 structural stability

**DOI:** 10.1093/bib/bbae400

**Published:** 2024-08-14

**Authors:** Benjamin Tam, Philip Naderev P Lagniton, Mariano Da Luz, Bojin Zhao, Siddharth Sinha, Chon Lok Lei, San Ming Wang

**Affiliations:** Faculty of Health Sciences, University of Macau, University Avenue, Taipa, Macau SAR 999078, China; Faculty of Health Sciences, University of Macau, University Avenue, Taipa, Macau SAR 999078, China; Faculty of Health Sciences, University of Macau, University Avenue, Taipa, Macau SAR 999078, China; Faculty of Health Sciences, University of Macau, University Avenue, Taipa, Macau SAR 999078, China; Faculty of Health Sciences, University of Macau, University Avenue, Taipa, Macau SAR 999078, China; Faculty of Health Sciences, University of Macau, University Avenue, Taipa, Macau SAR 999078, China; Faculty of Health Sciences, University of Macau, University Avenue, Taipa, Macau SAR 999078, China

**Keywords:** Ramachandran plot, molecular dynamics simulations, TP53, somatic missense variants

## Abstract

Somatic variation is a major type of genetic variation contributing to human diseases including cancer. Of the vast quantities of somatic variants identified, the functional impact of many somatic variants, in particular the missense variants, remains unclear. Lack of the functional information prevents the translation of rich variation data into clinical applications. We previously developed a method named Ramachandran Plot–Molecular Dynamics Simulations (RP-MDS), aiming to predict the function of germline missense variants based on their effects on protein structure stability, and successfully applied to predict the deleteriousness of unclassified germline missense variants in multiple cancer genes. We hypothesized that regardless of their different genetic origins, somatic missense variants and germline missense variants could have similar effects on the stability of their affected protein structure. As such, the RP-MDS method designed for germline missense variants should also be applicable to predict the function of somatic missense variants. In the current study, we tested our hypothesis by using the somatic missense variants in *TP53* as a model. Of the 397 somatic missense variants analyzed, RP-MDS predicted that 195 (49.1%) variants were deleterious as they significantly disturbed p53 structure. The results were largely validated by using a p53–p21 promoter–green fluorescent protein (GFP) reporter gene assay. Our study demonstrated that deleterious somatic missense variants can be identified by referring to their effects on protein structural stability.

## Introduction

Deleterious genetic variation causes functional defects of the affected genes and contributes to various types of human diseases including cancer. Deleterious genetic variation is also a valuable biomarker for clinical prevention, diagnosis, treatment and prognosis of related diseases. There are two types of genetic variation: somatic variation and germline variation. The somatic variation originates during the lifetime of the carrier and is present only in the affected cells and not heritable, whereas germline variation arose during the evolutionary process, is present in every cell of the carrier, and is inheritable across generations. The prevalence of somatic variation is much higher than germline variation in the affected genes and related diseases. For example, deleterious somatic variation in *EGFR* contributes to over 60% of nonsmall cell lung cancer [1], whereas deleterious germline variation in *BRCA1* and *BRCA2* associated with hereditary breast and ovarian cancer (HBOC) contributes only 5%–10% of breast cancer and 15% of ovarian cancer [2].

The rapid progress of genomic studies has led to the identification of a vast quantity of genetic variants. Knowing the functional impact of the variants is a pre-condition to translate the knowledge into clinical applications. Different approaches have been developed to determine the function of germline variation and somatic variation. The American College of Medical Genetics and Genomics / the Association for Molecular Pathology (ACMG/AMG) guidelines are widely used as a standard to classify germline variants [3]. It sets detailed criteria to classify a germline variant into one of the functional categories of Pathogenic, Likely Pathogenic, VUS (variants of unknown significance), Likely Benign, and Benign, and the ClinVar database is the largest database for the curated germline variants in different genes and diseases [4]. For somatic variation, however, there are less unified criteria in classifying somatic variation than in germline variation, and the current efforts are largely made in documentation rather than functional classification. For example, the COSMIC (Catalogue of Somatic Mutations in Cancer) database is currently the largest resource hosting the somatic variants identified in various types of cancer [5]. Under the scope of COSMIC, the Cancer Mutation Census (CMC) project ‘integrates all coding somatic mutations collected by COSMIC with biological and biochemical information from multiple sources, combining data obtained from manual curation and computational analyses’ (https://cancer.sanger.ac.uk/cmc/home). Under the CMC curation system, a somatic variant is classified into one of the five groups: ‘Tier One’, ‘Tier Two’, ‘Tier Three’, ‘Other mutations’, and ‘Synonymous mutations’. The variants in Tier 1, Tier 2, and certain variants in Tier 3 are considered to be similar to the ‘Pathogenic’ and ‘Likely pathogenic’ variants in germline variant classification, whereas these grouped in ‘Other mutations’ and ‘Synonymous mutations’ are not (https://cancer.sanger.ac.uk/cmc/help). Instead of making a conclusive classification on its own, the CMC classification system relies on the classification information made by the original submitters [5]. However, the classification from different sources are often highly redundant, inconsistent, and even controversial to each other, such that it is up to the users to make their own interpretation of whether a somatic variant is pathogenic or nonpathogenic. Compared to the germline variation, therefore, functional classification of somatic variants remains a big challenge. Furthermore, functional classification for somatic missense variation is even more troublesome as it doesn’t disrupt the translation of full-length protein but changes a single codon. The lack of conclusive classification for somatic variants leaves high uncertainty for their clinical relevance and usage. This is particularly important when considering the much higher prevalence of somatic variants than germline variants in human diseases.

We recently developed the RP-MDS (Ramachandran Plot–Molecular Dynamics Simulation) method for the functional classification of germline missense variants [6]. Molecular Dynamics Simulation (MDS) is a computation-based atomistic simulation method [7]. By analyzing the physical movement of atoms and molecules for a given time period, the trajectories generated by MDS are used to measure the macroscopic thermodynamic properties of the protein structure. Ramachandran Plot (RP) measures the rigidity of the N-C peptide bond [8]. By ruling out the unfavourable structure conformation due to the collusion between nonbonded atoms, RP provides high accuracy for the protein structure. The combination of MDS and RP provides a powerful tool to measure the effects of a genetic variant on protein structure, and the information is applicable to predict the deleteriousness of genetic variants. Using the RP-MDS method, we were able to predict the deleterious germline missense variants in multiple cancer genes, including *TP53*, *BRCA1,* and *BRCA2* [6, 9–11]. We reasoned that somatic missense variants and germline missense variants should have the same effects on local protein structure, regardless of their biological and evolutionary differences. As such, the RP-MDS method designed for characterizing germline missense variants should also be applicable to characterize the deleterious somatic missense variants.

*TP53* is a tumour suppressor gene. p53 functions as a tetrameric transcription factor to regulate the expression of the genes controlling cell cycle, apoptosis, metabolism, and genome stability [12]. *TP53* is also one of the most mutable genes in cancer, mostly missense variation in its DNA-binding domain (DBD). Deleterious missense variation can alter p53 structure and affect p53 binding to the targeted genes, leading to altered gene expression, genome instability, and oncogenesis [13, 14]. Deleterious *TP53* germline variation causes Li–Fraumeni syndrome, an autosomal dominant hereditary disease primed to various types of cancer [15], whereas *TP53* somatic variation occurs in almost all types of human cancer [16–19]. There were 3144 TP53 germline variants in the ClinVar database, of which 1263 (40.17%) were missense and 621 were VUS (https://www.ncbi.nlm.nih.gov/clinvar/?term=TP53[gene], accessed 15 January 2024). In comparison, there were 52 524 *TP53* somatic variants in the COSMIC database, of which 33 070 (62.96%) were missense without conclusive classification (https://cancer.sanger.ac.uk/cosmic/gene/analysis?ln=TP53#distribution, accessed 11 January 2024).

In the current study, we used *TP53* somatic missense variants as a model to test the potential of using RP-MDS to identify the deleterious somatic missense variants based on their impact on p53 structure. We identified a total of 397 *TP53* somatic missense variants from the COSMIC database. By using RP-MDS, we measured the impact of each variant on p53 structure stability. We predicted 195 (49.1%) of the variants as deleterious as evidenced by their distortion of p53 structure stability. Using an *in vitro* p53–p21 promoter–GFP reporter assay, we validated the reliability of the prediction. Our study demonstrates that deleterious somatic missense variants can be identified by directly referring to their impact on the stability of protein structure.

## Methods

### Sources of missense benign, pathogenic, and unclassified variants

From the COSMIC database, we identified 26 pathogenic missense variants in Tier 1 and Tier 2 and 18 unclassified missense variants under ‘Other mutations’ and used these to train the RP-MDS model. In order to balance the number of variants between Tier 1/Tier 2 and ‘Other mutations’, eight wildtype bases randomly selected from p53 DBD were also included to increase the number of variants under ‘Other mutations’. All variants selected in Tier 1 and Tier 2 were also present as pathogenic, and the variants in ‘Other mutations’ were also present as benign in the ClinVar database (https://www.ncbi.nlm.nih.gov/clinvar/, accessed April 9, 2020). We downloaded 29 900 *TP53* variants from the TP53 database (https://tp53.isb-cgc.org, R20 of July 2019, accessed 21 April 2021). After filtering the germline variants in ClinVar and by our previous study [9–11], and the duplicated variants, we identified 397 *TP53* somatic missense variants (Table S1).

### Molecular Dynamics Simulation and Ramachandran Plot

The process was performed following the detailed procedures [9]. In MDS, the MODELLER software in the Chimera package was used to build the missing atoms in the p53 DBD template (PDB ID: 2OCJ covering 94–313 residues, resolution 2.05 Å), and Rotamer software was used to replace the wildtype amino acid residues with the missense variant altered residues [20]. The altered p53 structure by the missense variant was used as the starting configuration for MDS in GROMACS (version 2021) [21]. The altered p53 structure was placed at the centre of the 10 × 10 × 10 nm simulation box, saturated with TIP3P water, and neutralized with Cl^−^ ions. AMBER03 force field was used to model the protein complex and ions. Steepest descent algorithms were applied to the system for 1 ns equilibration run at 298 K and 1 bar in the NPT ensemble using Berendsen thermostats and barostats algorithms. Afterwards, V-rescale thermostats and Parrinello–Rahman barostats were used for a 40 ns production run for the altered structure [22]. The trajectories from the last 10 ns production run were used for further analysis. A 2 fs timestep was set as the basis for the Verlet velocity algorithm. The particle mesh Ewald method was used to treat the long-range electrostatic potentials, and the cut-off distance was set at 1.0 nm.

The Ramachandran scatter plot (RSP) for each structure was generated by utilizing “gmx rama” command in the GROMACS software [21]. The RSP for each variable structure was generated from the last 10-ns of the MDS production run and converted into the Ramachandran density plot (RDP) by kernel density estimation using the Python “SciPy” module [23, 24]. A grid dimension of 32 × 32 was used for each RDP, and each grid point was used as a base for comparison.


(1) \begin{equation*} D(variant)=\left[\begin{array}{@{}c@{}}{\chi}_{t,1}\\{}\vdots \\{}{\chi}_{t,n}\end{array}\right]-\left[\begin{array}{@{}c@{}}{\chi}_{b,1}\\{}\vdots \\{}{\chi}_{b,n}\end{array}\right]=\left[\begin{array}{@{}c@{}}{\chi}_{d,1}\\{}\vdots \\{}{\chi}_{d,n}\end{array}\right] \end{equation*}

$$ where\ \left[\begin{array}{@{}c@{}}{\chi}_{b,1}\\{}\vdots \\{}{\chi}_{b,n}\end{array}\right]=\frac{\left[\begin{array}{@{}c@{}}{\chi}_{bV_1,1}\\{}\vdots \\{}{\chi}_{bV_1,n}\end{array}\right]+\cdots +\left[\begin{array}{@{}c@{}}{\chi}_{bV_i,1}\\{}\vdots \\{}{\chi}_{bV_i,n}\end{array}\right]+\left[\begin{array}{@{}c@{}}{\chi}_{W_1,1}\\{}\vdots \\{}{\chi}_{W_1,n}\end{array}\right]+\cdots +\left[\begin{array}{@{}c@{}}{\chi}_{W_j,1}\\{}\vdots \\{}{\chi}_{{W_j}_{,},n}\end{array}\right]}{i+j} $$

Each grid point was compared to the ${\chi}_{b,n-}$based file, which was comprised of an average of 18 benign variants, ${bV}_i$, and eight wildtype bases, ${W}_j$ RDP grid points, where $i\ and\ j$ are the number of benign and wildtype RDP.

A binary system (1, 0) was used to determine whether the difference between the variant and the based file grid point was beyond the standard deviation, $S$ of the average of 18 benign variants and wildtype bases. The following equation illustrated the determination of the binary “1” or “0”:


(2) \begin{equation*} G=\frac{\sum_n^1\left\{\begin{array}{@{}c}b=1, if\ {\chi}_{d,n}>S\ \\{}b=0, if\ {\chi}_{d,n}\le S\end{array}\right.}{n} \end{equation*}

The natural log of the total grid point, $G$ binary system (in percentage) was used to determine the deleteriousness of the variant. ‘Deleterious’ or ‘non-deleterious’ was used to classify somatic variants.

An open RP-MDS server “RP-MDS for classification of TP53 missense variants” is developed for online TP53 missense variant analysis (https://genemutation.fhs.um.edu.mo/rp-mds).

### GFP-based functional assay

A GFP-based reporter assay was applied to validate the predicted deleterious effects of somatic missense variants on the regulation of gene expression [19, 25]. The assay consisted of two key components: the p53 expression plasmid pLX313-TP53 and the p53 binding plasmid pGL2-p21-promoter-GFP [25]. The variant-modified p53 expressed from pLX313-p53 binds to the p53 binding motif in the p21-promoter of pGL2-p21-promoter-GFP to regulate GFP expression, which reflects the impact of variant-modified p53 on its binding ability to the p53 binding motif in p21-promoter [26]. The plasmid pLX313-TP53 containing wildtype TP53 was used as the normal control. Single-base variants were introduced into TP53 in pLX313-TP53, in which the variant-containing fragments were generated by polymerase chain reaction (PCR) using the primers containing the variants and used to replace the wildtype TP53 in the pLX313-TP53 by restriction digestion and ligation, transformation, and purification. Each variant-containing TP53 construct was confirmed by Sanger sequencing. The criteria for variant selection were as follows: (i) the benign and pathogenic variants were the somatic and germline variants annotated in both COSMIC database and ClinVar database. (ii) the deleterious variants predicted were also the somatic and germline databases present in COSMIC database and ClinVar database. (iii) the variants were predicted by RP-MDS as deleterious for both germline model and the somatic model. The following variants were selected for the validation test: (i) benign variants: c.319 T > C p.Y107H, c.385G > A p.A129T, c.704A > G p.N235S; (ii) pathogenic variants: c.733G > A p.G245S, c.817C > T p.R273C; and (iii) predicted deleterious variants: c.293C > T p.P98L, c.296C > T p.S99F, c.319 T > G p.Y107D, c.346 T > G p.S116A, c.349G > A p.G117R, c.373A > C p.T125P, c.405C > G p.C135W, c.427G > T p.V143L, c.458C > T p.P153L, c.505A > G p.M169V, c.641A > C p.H214P, c.656C > G p.P219R, c.758C > A p.T253N, c.764 T > G p.I255S, c.773A > G p.E258G, c.785G > T p.G262V, c.820G > C p.V274L, and c.833C > G p.P278R.

The H1299 TP53^−/−^ epithelial-like cell line was used to assess the effects of the variant-containing TP53 on gene expression by cotransfection of variant-modified pLX313-TP53 and pGL2-p21-promoter-GFP plasmids. 1 × 10^4^ H1299 cells in RPMI1640 medium with 10% fetal bovine serum were cotransfected with 100 ng p53 plasmids and 100 ng p21-promoter-GFP plasmids using lipofectamine 3000 Transfection Reagent (Thermo Fisher Scientific, MS, USA). Forty-eight hours after the transfection, GFP activities in the transfected cells were read using the PerkinElmer Victor X3 Micro-plate Reader. The relative fluorescence value (RFU) was used to define p21 promoter activities in the H1299 cells and subtracted by the value from the negative control (cells transfected with no expression plasmids) to obtain the final RFU. Three repeats were performed for each test, and the values were averaged and presented as mean ± SEM. One-tailed *T*-test was used to compare the RFU between the wildtype and variant-containing TP53, with *P*-value <.05 considered as deleterious and *P*-value >.05 as non-deleterious. Statistical analyses were performed using Prism 9 software (GraphPad).

## Results

### The degree of germline and somatic missense variants altering the same codon

Using the DNA damage repair (DDR) genes as the model, we analyzed the degree of germline variants and somatic variants causing the same codon change by referring to the germline variants in ClinVar and somatic variants in COSMIC databases. In the 170 DDR genes [27], we identified a total of 128 657 germline variants and 45 271 somatic variants, of which 12 421 were shared between germline and somatic accounting for 11% of the germline variants and 27% of somatic variants (Table S2). For example, 658 (78%) of the 841 *PALB2* somatic variants, 922 (62%) of the 1495 *TP53* somatic variants, and 989 (61%) of the 1634 *BRCA2* somatic variants were shared with the germline variants in the same genes (Table 1). The data highlight that somatic and germline missense variants altering the same codon are widely present, particularly in functionally important genes as represented by DDR genes.

**Table 1:** Examples of DDR genes with highly shared germline and somatic missense variants.

Gene	Germline (ClinVar)	Somatic (COSMIC)	Common	Common rate (%)	
				Germline	Somatic
PALB2	2708	841	658	24.3	78.2
MSH6	4394	716	549	12.5	76.7
MSH2	2782	483	363	13.0	75.2
MLH1	1984	382	263	13.3	68.8
RAD51C	907	142	93	10.3	65.5
ATM	7213	2015	1308	18.1	64.9
TP53	1275	1495	922	72.3	61.7
BRCA2	8172	1634	989	12.1	60.5
BARD1	1941	369	218	11.2	59.1
PTEN	961	836	491	51.1	58.7
POLE	4302	1175	648	15.1	55.1
BRIP1	2626	536	295	11.2	55.0
NBN	1580	331	175	11.1	52.9
PMS2	2493	532	277	11.1	52.1
RAD50	2255	487	252	11.2	51.7
MSH3	2231	374	186	8.3	49.7
BLM	2144	561	266	12.4	47.4
MRE11	1012	294	133	13.1	45.2
BRCA1	5425	916	400	7.4	43.7
FANCA	1538	638	274	17.8	42.9
XRCC2	375	115	48	12.8	41.7
RNASEH2A	182	85	34	18.7	40.0
MLH3	1499	398	159	10.6	39.9
FANCC	746	235	92	12.3	39.1
POLD1	2036	540	200	9.8	37.0
ERCC2	806	404	148	18.4	36.6
SLX4	1170	656	229	19.6	34.9
FANCE	279	137	44	15.8	32.1
RAD54L	599	252	79	13.2	31.3
FANCM	1361	681	192	14.1	28.2
FANCI	738	370	98	13.3	26.5
FANCF	232	126	31	13.4	24.6

### Determination of threshold between deleterious and nondeleterious variants

**Figure 1:**
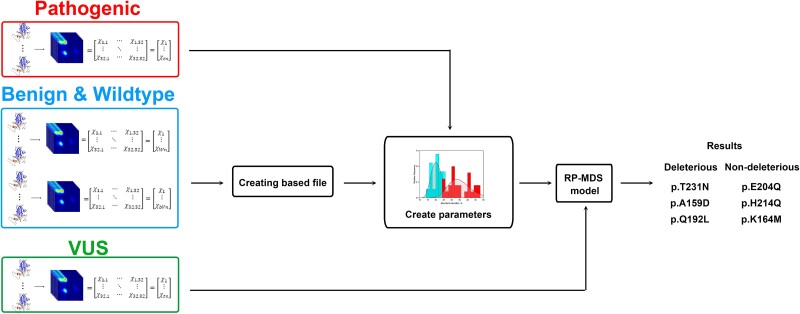
Outline of the RP-MDS process. RDP from benign variants and wildtype bases were combined to create the based file, and used to compare with the RDP from pathogenic variants and create the parameters to classify somatic missense variants into deleterious or nondeleterious categories.

We identified the existing pathogenic variants, benign variants, and wildtype bases located in p53 DBD and used those as the training materials to set the threshold to differentiate deleterious and nondeleterious variants. The Ramachandran density plot (RDP) from benign variants and wildtype were combined to create the based files and used to compare with the RDP of pathogenic variants. Figure 1 shows the flowchart of RP-MDS process.

**Figure 2:**
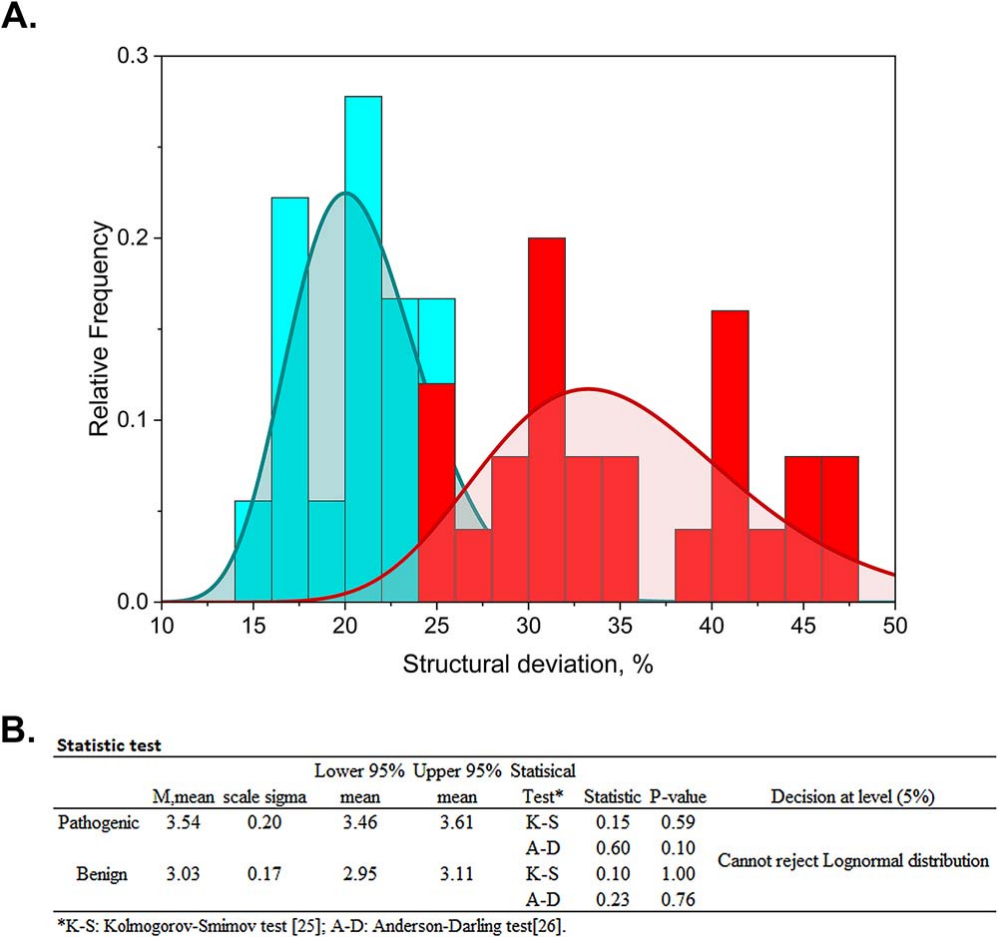
Structural deviation distribution of benign and pathogenic variants. (A) Relative frequency of structural deviation between benign variants and pathogenic variants. The graph shows the collective structural deviation of benign and pathogenic variants, and log-normal distribution curves were fitted against the data. (B) Statistic data showing the distribution. Teal: benign variants; red: pathogenic variants.

To set the cut-off thresholds for non-deleterious and deleterious variants, we first performed MDS for the 25 pathogenic variants, 18 benign and 8 wildtype alleles. The known benign and pathogenic missense variants used to train RP-MDS affect p53 codons of G105, C141, A159, A161, H168, E171, C275, and P278. They were also germline variants present in ClinVar database. RSP was extracted from the last 10 ns MDS trajectories, converted to the RDP and used to calculate the structural deviation for benign and pathogenic variants (Fig. 2A). Kolmogorov–Smirnov (K-S) and Anderson–Darling (A-D) statistical tests showed that the data did not reject log-normal distribution (Fig. 2B) [28, 29]. The results from a two-sided *t*-test showed that the mean values between the benign and pathogenic data were significantly different (*P* < .0001), indicating that the benign and pathogenic missense variants had distinct effects on p53 structure. Based on the pathogenic log-normal distribution curve, we set the structural deviation >3.46 as deleterious and ≤3.46 as non-deleterious.

### Classification of somatic missense variants

**Figure 3:**
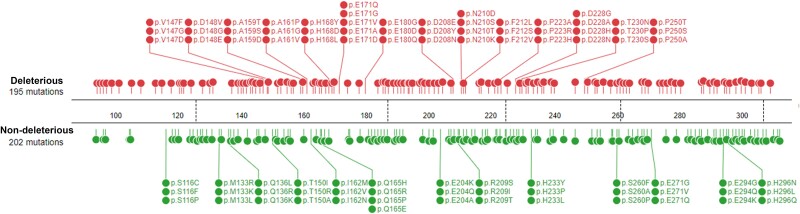
Classification of 397 TP53 somatic missense variants. The lollipop graph shows the positions and frequencies of the RP-MDS-predicted deleterious and non-deleterious somatic missense variants in p53 DBD region. Green: non-deleterious variants; red: deleterious variants.

**Figure 4:**
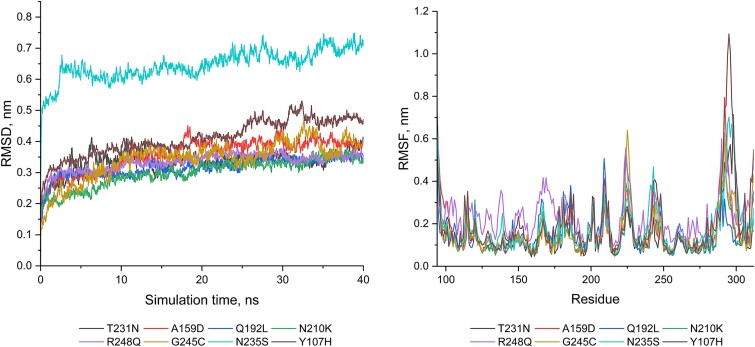
MDS trajectories of TP53 deleterious somatic missense variants. Left: RMSD; right: RMSF of the deleterious variants (p.T231N, p.A159D, p.Q192L, p.N210K), the known pathogenic variants (p.R248Q and p. G245C), and the known benign variants (p.N235S and p.Y107H).

**Figure 5:**
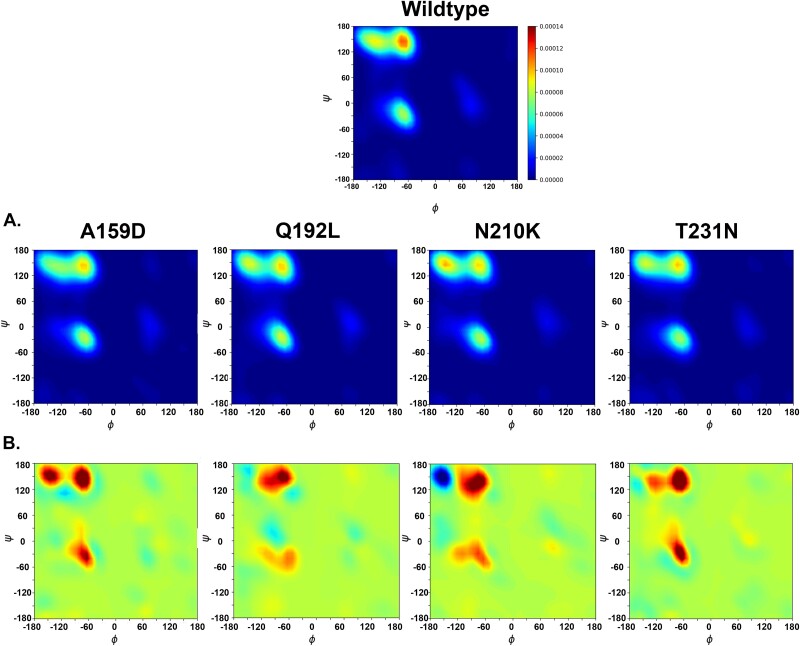
Ramachandran density plot of TP53 deleterious somatic missense variants. (A) RDPs for deleterious variants (p.A159D, p.Q192L, p.N210K, and p.T231N). The colours from blue to red represent low to high density. (B) The differences between the variants and the based files. The colours from blue to red represent from diminished to increased density.

We identified 397 unclassified somatic missense variants. Of the 397 variants, 384 (96.7%) were classified as ‘other mutations’, 11 (2.8%) as Tier 3, 1 (0.25%) as Tier 2, and 1 (0.25%) had no classification by COSMIC database. Using the wildtype p53 as the template, we generated the variable p53 structures for the 397 unknown somatic missense variants. We performed MDS and RP to classify the variants. Under the cut-off threshold at >3.46, we observed that 195 (49.1%) variants had a deleterious impact on the p53 structure (Table S3). These variants were enriched at the residue positions of G105, C141, P153, A159, A161, H168, E171, E180, D186, D208, N210, P223, D228, C275, and P278. Figure 3 shows the distribution and frequency of the residues altered by the variants in p53. Figure 4 shows the representative distribution of a group of variants measured by RMSD and RMSF in MDS, which indicated the likelihood of deleteriousness for the classified variants [9]. The predicted deleterious variants were concentrated more at the 150–170 and 200–240 residue regions, whereas the predicted non-deleterious variants were concentrated more at the 130–160, 210–230, and 260–300 residues regions (Suppl. Fig. 1A). However, the difference between the deleterious and non-deleterious data was not significant (Fig. S1).

p.A159D, p.Q192L, p.N210K, and p.T231N had the highest deleterious impacts on TP53 structure as reflected by their high structural deviations in the range of 3.88–3.91, corresponding to 48.3%–49.9% of structural change. Figure 5 shows the distribution of RP images. Using the last 10 ns of MDS trajectories, it showed the overall protein structural change by quantifying the protein backbone (φ and ψ) for each residue (Fig. 5A). Each variant was compared to the based files from 18 benign variants and seven wildtype bases. The results showed that for all predicted deleterious variants, the density peaks for the α-helix region [φ, ψ = (−60, −30)], PII-spiral regions, and β sheet region significantly increased, reflecting their significant deviation from the benign and wildtype structures (Fig. 5B), evidencing the deleterious impact of the missense variants on p53 structure stability.

**Figure 6:**
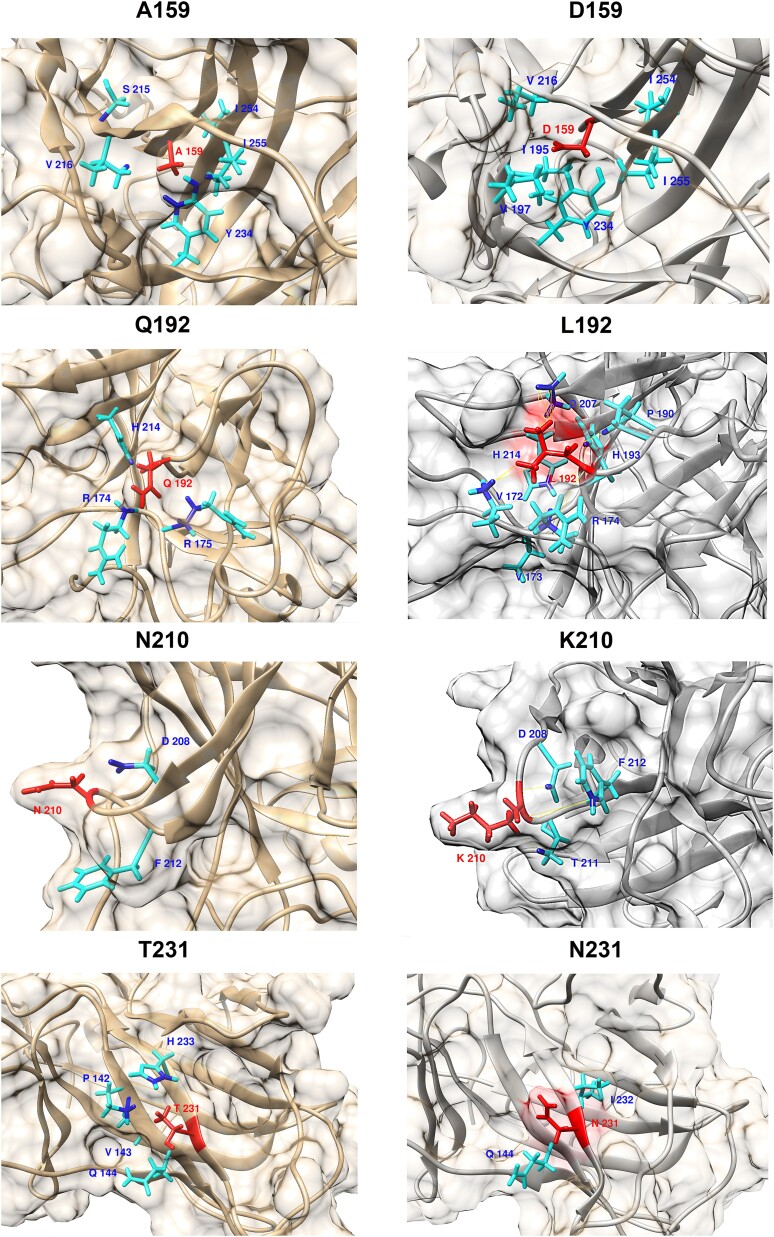
Examples of the deleterious somatic missense variants on p53 structure. Left: wildtype residues; right: altered residues. The figure shows the altered interactions between the altered residues (p.A159D, p.Q192L, p.N210K, and p.T231N) and their neighbour amino acid residues. Red: amino acid residue changed by the variants; teal: interaction between residues.

Figure 6 further shows the interactions of each altered residue with their local amino acids residues:

- Wildtype A159 interacted with S215, V216, Y234, I254, and I255 to maintain the β sheet structure, whereas the altered residue D159 altered the interaction with I195, V197, V216, Y234, I254, and I255, which destabilized the β strand and led to additional bend on residue 165–169;
- Wildtype Q192 interacted with R174, R175, E180, and H214 to maintain the α-helix in the L2 loop, whereas the altered residue L192 altered the interaction to additional residues V172, V173, P190, H193, and D207 and destabilized the α-helix in the L2 loop;
- Wildtype N210 interacted with F212 and D208 to maintain the bend of the β-sheet, whereas the altered residue K210 interacted with T211 causing destabilization of β-strand S6 and S7 through greater flexibility, besides with F212 and D208;
- Wildtype T231 interacted with V143, T230, and I232 to maintain a stable β-strand, whereas the altered residue N231 interacted additionally with D144, causing the β-sheet to bend away from the structure core.

**Figure 7:**
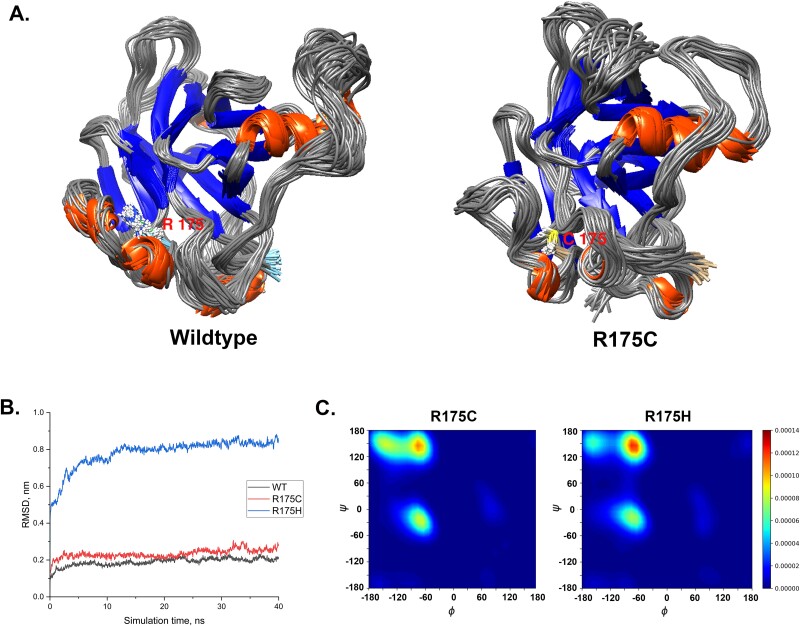
Comparison of p.R175C, p.R175H, and wildtype structure. (A) MDS-superimposed structure of wildtype and p.R175C; (B) RMSD trajectories of wildtype, p.R175C, and p.R175H by MDS. (C) RDPs of p.R175C and p.R175H.

The function of multiple TP53 hotspot variants affecting codon R175 and G245 remains undetermined. For example, R175C didn’t affect cell cycle arrest and apoptosis [26–28]. RP-MDS results showed that the overall structure fluctuation of R175C was similar to the wildtype structure (Fig. 7A), and the root mean square deviation (RMSD) of R175C, R175H, and wildtype also showed that R175C had similar RMSD to the wildtype one (Fig. 7B). However, the RP of R175C and R175H showed significant differences in the PII-spiral regions [φ, ψ = (−60, +150)] and β sheet region [φ, ψ = (−120, +130)] (Fig. 7C). As measured by the cut-off thresholds, R175C had a low structural deviation of 28% whereas R175H had a high structural deviation of 41% (Table S3). Therefore, RP-MDS provided structural evidence indicating that R175C is non-deleterious whereas R175H is deleterious.

### Functional validation for the predicted deleterious variants

**Figure 8:**
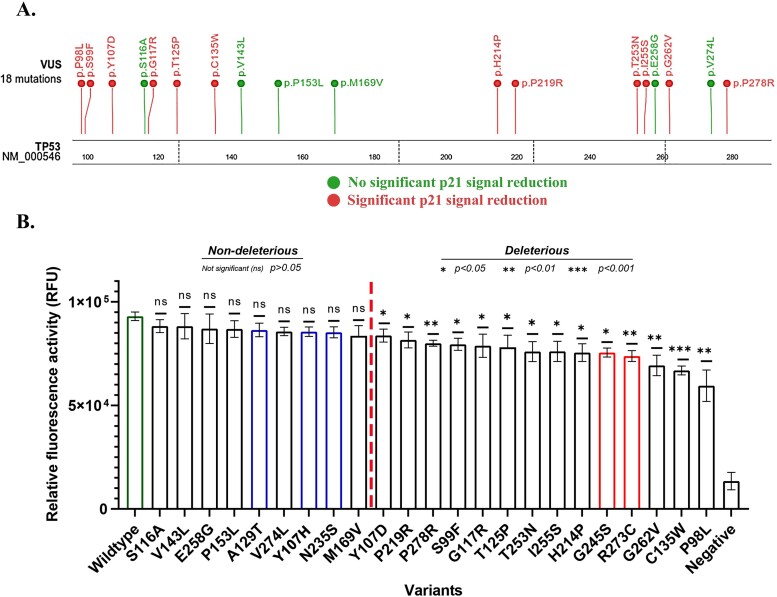
Validation of the predicted deleterious somatic missense variants on gene expression regulation. (A) Location of the predicted deleterious variants along p53 DBD region. Red: significantly reduced GFP expression; green: nonsignificant changed GFP expression. (B) Comparison of GFP expression between benign, pathogenic, and predicted deleterious variants. The variants right of the red dot line had significantly reduced GFP expression. Blue: benign; red: pathogenic.

We used a p53–p21 promoter–GFP reporter assay to validate the predicted deleterious somatic missense variants on gene expression. In the assay, the expressed p53 in pLX313-TP53 bound to the p53 binding motif in p21 promoter to control the expression of GFP in pGL2-p21-promoter-GFP [25]. The missense variant-containing pLX313-TP53 constructs were co-transfected with the pGL2-p21-promoter-GFP in H1299 TP53^−/−^ cell line. By using the wildtype p53 as the control, the changed GFP level represented the effects of the altered p53 on gene expression regulation. We tested a total of 23 variants including three benign and two pathogenic controls and 18 RP-MDS predicted deleterious somatic missense variants. The results showed that 12 of the 18 (66.7%) predicted deleterious variants significantly reduced GFP expression, including the variants for S99F, Y107D, G117R, T125P, H214P, P219R, T253N, and I255S variants (*P* < .05), P98L, G262V, and P278R (*P* < .01), and C135W variant (*P* < .001) (Fig. 8). Of the six tested variants showing no significant changes, P153L and M169V were located in the p53 DBD region without defined secondary structure.

## Discussion

Determination of the deleteriousness of somatic variation remains a challenge. Using the somatic missense variants in TP53 as a model and the RP-MDS as the tool, our study shows that deleterious somatic missense variants can be identified by referring to their impact on protein structural stability.

The RP-MDS method uses protein structure as the reference to determine the function of missense variants. It avoids many uncertainties often present in other means. For example, the evidence listed by the CMC/COSMIC is largely originated from other *in silico* prediction methods, of which many often use evolution conservation-based sequence homology to determine the function of somatic variants. However, somatic variants are not heritable. As such, the same codon changes by the same somatic variants across different species would not be possible except these occurred by coincidence. In contrast, RP-MDS uses protein structure as the only reference. Protein structure is determined by intramolecular interactions of electrostatic, hydrophobic, hydrogen bonding, and Van der Waals interactions. During the RP-MDS process, MDS relaxes and equilibrates the dynamic conformational changes of protein structure by following the Newtonian equation of motions for a given simulation period [30, 31]; RP then uses the MDS trajectories to precisely quantify the macroscopic properties of the protein structure by referring to the torsion angles of the N-C peptide bond restricted by sterically unfavourable structure conformation. Therefore, RP-MDS can precisely detect the effects of missense variants on protein structure [8, 9, 11, 32].

In our previous studies, we compared extensively between RP-MDS and other *in silico* methods for variant classification (Table 1B in [6]; Table 1B in [9]; Table 2 and Fig. 5 in [11]). The results from these studies clearly show that RP-MDS is superior to each method compared in many ways. For example, in the TP53 germline missense variant study [9], we compared the classification of 340 germline missense VUS between RP-MDS and 10 *in silico* methods of Polyphen2_HDIV, Polyphen2_HVAR, SIFT, M-CAP, MutationTaster, LRT, PROVEAN, FATHMM, MetaSVM, and MetaLR. The results showed that RP-MDS had the lowest rate for the ‘deleterious’ classification and the highest rate for the ‘undefined’ classification among all methods tested (Table 1B in [9]), demonstrating that RP-MDS provides high specificity to classify germline missense variants. Low specificity is an inherited problem in current *in silico* prediction methods that they ‘tend to have low specificity, resulting in over-prediction of missense changes as deleterious and are not as reliable at predicting missense variants with a milder effect’ as indicated by the ACMG/AMG guidelines [3]. Our current study indicates that the high specificity of RP-MDS demonstrated in germline variant analysis is also present in somatic variant analysis. This is due to the fact that the structural instability by the germline and somatic variants causing the same codon change is similar.

The cut-off to differentiate deleterious and non-deleterious variants is based on the statistical differences between the pathogenic variants and the benign variants [6]. We used the 95% percentile, or 3.46, of the lower mean to compensate for possible statistical error. We postulated that although most of the pathogenic missense variants would have impact on protein structure, not all pathogenic missense variants will cause structural change. These could be ignored by RP-MDS. Therefore, the threshold can be arbitrary in affecting these slightly above or below the threshold. Therefore, the threshold for different studies may need to be adjusted to minimize its effects on certain variants.

Under the selection criteria, only 26 pathogenic and 18 benign missense variants and eight wildtype alleles were used for the training. Those might seem to be a small sample size. However, each variant had 333 conformation points and each conformation was saved every 30 ps over the 10 ns sampling period. The total combined points from the training process reached 17 316 [(26 pathogenic + 18 benign + 8 wildtype alleles) × 333]. The rich information allows RP-MDS to distinguish the conformation between deleterious and non-deleterious structure. However, the information may not cover all possible deleterious conformation. When possible, increasing pathogenic and benign variants in the training model to provide more deleterious confirmations may provide better distinction between deleterious and non-deleterious structures. Further, the running time for MD simulation may also be a factor to consider. Different studies may use different simulation time; for example, 30 ns was used in a study [33]. Our study used 40 ns. As long as computational power allows, longer time may provide better equilibrates and therefore improve the accuracy of detection. We also the following measures to ensure the accuracy of classification: (i) used known pathogenic variants as control to see if these can be predicted as deleterious; (ii) used known benign variants as control to see if these can be predicted as non-deleterious; and (iii) used GFP reporter gene assay to validate if the predicted deleterious variants can affect gene expression.

The deleterious variants classified by RP-MDS should cause significant change of TP53 structure, as defined by the classification criteria. However, in the validation test, 6 of the 18 tested deleterious variants did not show significant GFP signal reduction (Fig. 8). Possible explanations could be (i) The test was performed only in a single cell line, which may not reflect the deleterious impact of the variants in other lineages due to the possible lineage specificity. (ii) The GFP expression relies on the interactions between the TP53–P21 promoter. The expression level of the altered p53 could be affected by the variants, causing lower presence of the altered p53. (iii) The transient co-transfection of altered p53 and p21 constructs may not allow synergistical expression of the proteins.

The structure–function relationship is one of the essential issues in TP53 variation [12, 13]. Deleterious variants can disturb p53 structural stability at different degrees from low distortion to global unfolding [14]. For example, R175 is located at the zinc-binding site and the DNA-binding interface, variants such as R175H can destabilize the structure, causing the loss of p53 transactivation and tumour-suppression function and the gain of oncogenic function under certain situations [34]. In contrast, R175C causes no structural change and therefore retains the function of cell cycle regulation as the wildtype p53 [18]. Another example is R249S located at the S4-S5 turn of p53. It distorts the alpha helix loop, leading to the disruption of the DNA binding and tumour-suppression function of p53 [19].

RP-MDS may also be applicable to classify the somatic variants in other somatically highly mutated tumor suppressors, oncogenes, and DDR genes (Table 1, Table S1). Taking *BRCA1* as an example: germline pathogenic variation in *BRCA1* is traditionally considered as the major contributor for *BRCA1*-related oncogenesis. A total of 400 (44%) of the 916 somatic variants in *BRCA1* were the same as germline variants (Table 1). The rich somatic variation data in *BRCA1* suggest that somatic variation in *BRCA1* may also play important roles in *BRCA1*-related oncogenesis. Applying RP-MDS to determine functional significance of the somatic variants in *BRCA1* may enhance our understanding of *BRCA1*-related oncogenesis.

While our study shows that somatic and germline missense variants can change protein structure, this similarity may not be interpreted automatically as they have the same function, even for the somatic and germline missense variants causing the same codon change, as the biology behind the germline variation and the somatic variation is very different: (1) Different mechanisms: a germline variant is inherited and is present in every cell of the carrier and has functional impact from fertilization across the entire lifetime, whereas a somatic variant occurs during life process of the carrier and is not inheritable; (2) Different diseases: the same germline and somatic variant can cause different diseases. For example, germline p53R175H is a genetic predisposition for Li–Fraumeni syndrome [34] but somatic p53-R175H is present in multiple cancer types including breast, lung, colorectal, and pancreatic cancers [35]; (3) Different timing of diseases caused: germline variation–related diseases usually occur at young age, whereas somatic variant–related diseases often occur at a later stage of life; (4) Different arising time: the germline variant in humans can be originated up to a thousand years ago [36], whereas the somatic variant is generated in a specific tissue/cell type during lifetime; and (5) Different heritage: a germline variant can be shared in multiple family members following the principles of Mendelian genetics, whereas a somatic variant is only present in individual carrier.

In conclusion, our present study shows that deleterious somatic missense variants can be identified by referring to their impact on the stability of protein structure, and the RP-MDS method can be a powerful means to identify the deleterious somatic missense variants.

**Key Points**

- The functional impact of many somatic missense variants remains unclear.
- We applied the Ramachandran Plot–Molecular Dynamics Simulation method to predict the deleteriousness of *TP53* somatic missense variants based on their effects on p53 structure stability.
- Of the 397 *TP53* somatic missense variants tested, we identified 195 (49.1%) variants as deleterious.
- The results were experimentally validated by a reporter gene assay.

## Supplementary Material

Figure_S1_bbae400

Table_S1_bbae400

Table_S2_bbae400

Table_S3_bbae400

## Data Availability

The data underlying this article are available at the RP-MDS server RP - MDS for classification of TP53 missense variants (https://genemutation.fhs.um.edu.mo/rpmds).
