# Supplementary figures and images for: Comprehensive classification of TP53 somatic missense variants based on their impact on p53 structural stability
Source: Brief Bioinform. 2024 Aug 14;25(5):bbae400. doi: 10.1093/bib/bbae400 (PMC11323084; doi:10.1093/bib/bbae400)

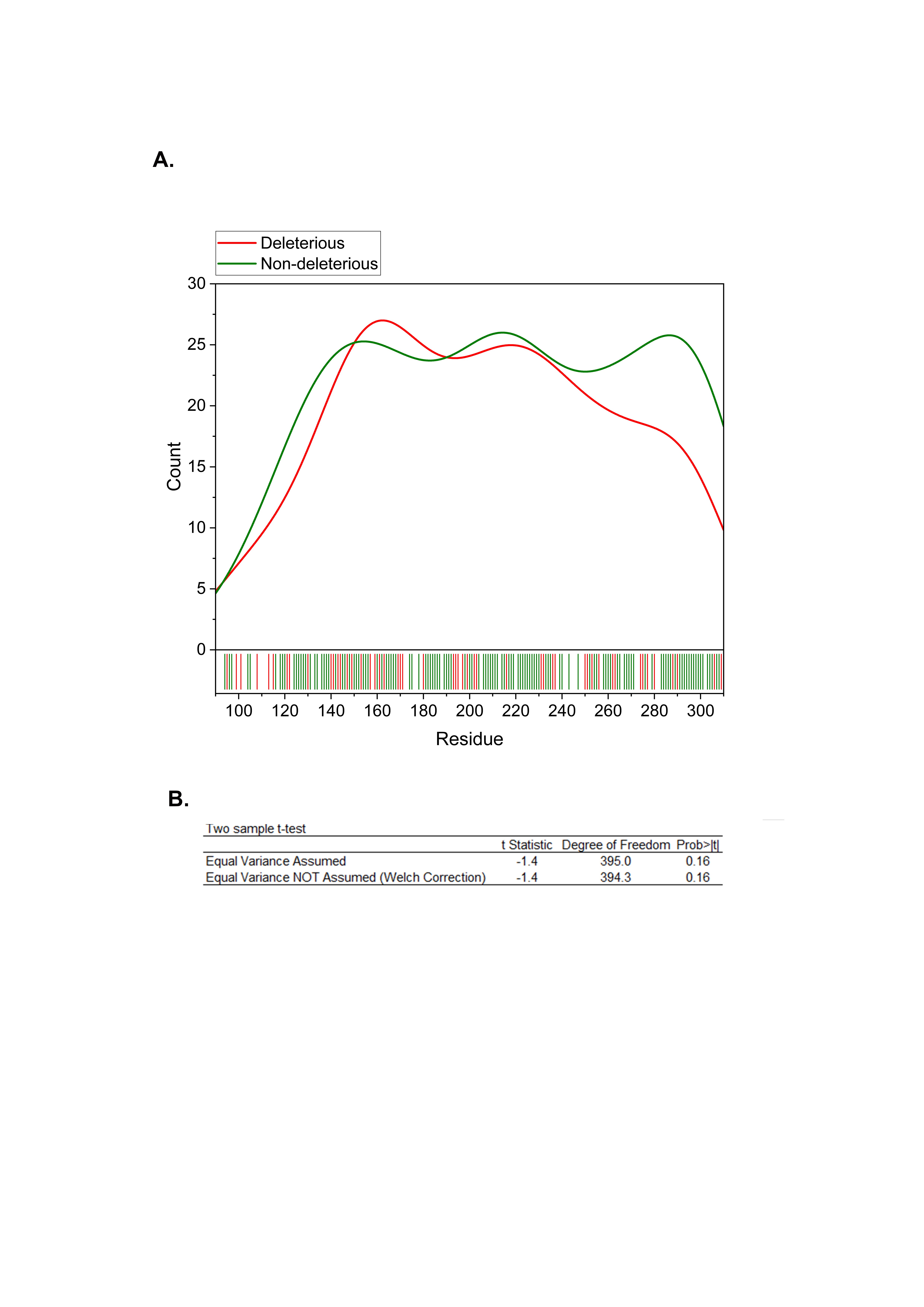

Supplement: Figure_S1_bbae400 [file figure_s1_bbae400.jpeg]
